# The combination of transarterial chemoembolization and microwave ablation is superior to microwave ablation alone for liver metastases from colorectal cancer

**DOI:** 10.1007/s00432-024-05951-8

**Published:** 2024-10-01

**Authors:** Thomas J. Vogl, Hannah Stefan, Tatjana Gruber-Rouh, Jörg Trojan, Wolf Otto Bechstein, John Bielfeldt, Hamzah Adwan

**Affiliations:** 1 Clinic for Radiology and Nuclear Medicine, University Hospital Frankfurt, Goethe University, Theodor-Stern-Kai 7, 60590 Frankfurt Am Main, Germany; 2 Department of Gastroenterology, Hepatology and Endocrinology, University Hospital Frankfurt, Goethe University, Theodor-Stern-Kai 7, 60590 Frankfurt, Germany; 3 Department of General and Visceral Surgery, University Hospital Frankfurt, Goethe University, Theodor-Stern-Kai 7, 60590 Frankfurt, Germany

**Keywords:** Liver metastases from colorectal cancer, Interventional radiology, Transarterial chemoembolization, Microwave ablation

## Abstract

**Objectives:**

This study aimed to compare the combination therapy of transarterial chemoembolization (TACE) and microwave ablation (MWA) with MWA alone in treating liver metastases from colorectal cancer (LMCRC).

**Materials and methods:**

In this retrospective study, a total of 251 patients with unresectable and not to chemotherapy responding LMCRC were included. Group A consisted of 184 patients (104 male and 80 females; mean age: 64 ± 11.4 years) with 442 metastases who received a combination of TACE and MWA. A total of 67 patients (49 male and 18 females; mean age: 63.2 ± 11.8 years) with 173 metastases patients were included in group B, who received only MWA. Parameters assessed were local tumor progression (LTP), hepatic distant tumor progression (hDTP), hepatic progression-free survival (hPFS), and overall survival (OS).

**Results:**

The rate of LTP was 4.9% in group A and 4.5% in group B (p-value: 0.062). The rate of hDTP was 71.7% and 83.6% for groups A and B (p-value: 0.81), respectively. The mean hPFS was 13.8 months (95% CI 10.9–16.8) for group A and 8.1 months (95% CI 6.1–10.1) for group B (p-value: 0.03). The median OS time for group A was 30 months (95% CI 26–34), with 1-, 2-, 3-, and 4-year OS rates of 84.2%, 61.1%, 40.8% and 31.3%, respectively. In group B however, the median OS time was 26 months (95% CI 18–34) with 1-, 2-, 3-, and 4-year OS rates of 82.3%, 53.2%, 34.6% and 28.2%, respectively (p-value: 0.67).

**Conclusion:**

The combination therapy of TACE and MWA is superior to the monotherapy of MWA for LMCRC, especially regarding hDTP, hPFS and OS.

## Introduction

Colorectal cancer (CRC) is the third most prevalent malignancy in both genders in Germany (Robert-Koch-Institut 2019), despite the fact that the Incidence has been decreasing due to screening using colonoscopy (Eickhoff et al. 2018). CRC takes the second place worldwide in cancer mortality, after cancer of the lungs (Bray et al. 2024).

The liver is the most frequently affected organ by metastases from CRC (Thelen et al. 2007). Surgical resection represents the treatment of choice for these liver metastases from CRC (LMCRC) (Thelen et al. 2007). Therefore, patients with unresectable hepatic lesions must be treated differently. Well-established approaches are minimally invasive local treatments, such as microwave- (MWA) or radiofrequency ablation (RFA) (Clark and Smith 2014). These treatments are heat-based and lead to tumor necrosis (Izzo et al. 2019; Gruber-Rouh et al. 2016). In comparison to RFA, MWA has a less prominent heat sink effect and produces larger ablation zones (Minami et al. 2023; Brace 2009).

Another possibility is the intravascular application of therapeutics, such as transarterial chemoembolization (TACE), in which chemotherapeutic and embolising substances are directed into the main vascular supply of the liver tumor through the use of catheters (Kwan and Pua 2021). There are several TACE techniques including conventional TACE (cTACE) and irinotecan-loaded drug-eluting beads TACE (DEBIRI-TACE) (Vogl and Lahrsow 2022). Advantages of TACE include higher local concentration of chemotherapeutic drugs within the tumor in comparison to systemic chemotherapy and that the cytotoxic effect is limited to the tumor. Healthy tissue is spared and side effects of the applied chemotherapeutic drugs reduced, making it better tolerated by patients (Massmann et al. 2015).

Several studies have shown the high efficacy of the combination of TACE and thermal ablation for hepatocellular carcinoma (HCC) (Ren et al. 2019; Wang et al. 2019; Yang et al. 2022). Therefore, this study shall analyze whether a combination of TACE and MWA could result in a better outcome for patients with unresectable and not to chemotherapy responding LMCRC in comparison to MWA alone.

## Materials and methods

### Study design

This study was approved by the university hospital’s ethics committee, and informed consent was obtained from each patient before the procedure.

We retrospectively reviewed the data from 251 patients with 615 LMCRC treated at our institution by TACE followed by MWA (Group A) or MWA alone (Group B).

TACE was performed prior to MWA, especially in highly hypervascularized lesions in order to devascularize the metastases, which leads to reduction of tumor burden as well as a reduction in complications such as hemorrhage during ablation (Vogl et al. 2003). Each patient’s case was previously discussed in a multidisciplinary tumor board to evaluate the best possible treatment options.

Patients were included in the study if the following criteria were fulfilled: 1. Patient above 18 years of age, with technically or due to the general condition of the patient unresectable and not to chemotherapy responding LMCRC; 2. Recent MRI studies available; 3. Treatment of the LMCRC by MWA with or without previous TACE 4: A minimum of one follow-up MRI studies performed; 5. No more than 5 liver metastases. Exclusion criteria were as follows: 1. Liver metastasis of primary tumors other than CRC; 2. No follow-up images available; 3. Size of LMCRC > 5 cm.

The included cases of patients in both groups were mainly evaluated according to age, sex, number and maximum axial diameter of metastases, number of performed treatments, maximum axial diameter of the ablation zone 24-h post-ablation, applied energy during MWA, duration of MWA, complications, complete ablation, local tumor progression (LTP), hepatic distant tumor progression (hDTP), overall survival (OS) and hepatic progression-free survival (hPFS).

### Measurements

The diameter of tumors was calculated based on the previous MR imaging of the patient’s abdomen. First post-ablation images were acquired 24 h after therapy, to evaluate the success of ablation therapy, as well as to measure the size of the ablation zone. The tumor ablation safety margin was evaluated utilizing the technique mentioned by Wang et al. (2013), by measuring the minimum distance of the index tumor to the boundary of the ablation zone based on the pre-ablation images and the 24 h post-treatment images. Anatomical landmarks in different directions were set in each pair of images and then used to obtain the lowest safety margin for the ablation procedure (Wang et al. 2013). Complete ablation (A0) was assumed if the target lesion was completely necrotized on the 24 h post-MRI scan with an ablative margin of ≥ 5 mm. In the case of an incomplete tumor ablation, a subsequent second ablation session was performed.

### Ablation procedure

The ablation procedure was planned based on the most recent available contrast-enhanced MRI scans. The index lesion was identified, the most ideal placement for the ablation antenna was determined, and the entry point was marked with Radiopaque markings on the patient's skin. The patients were monitored during the entire procedure via blood pressure, pulse oximetry, and electrocardiography.

Before the beginning of the procedure, the patients were administered a combination of a sedative and analgesic medication consisting of diazepam (Diazepam-ratiopharm®, ratiopharm GmbH) in a dosage of 0.1–0.2 mg/kg body weight and piritramide (Piritramid-hameln®, Hameln Pharma Plus GmbH) in a dosage of 0.2 mg/kg body weight.

For the ablation procedure, we used the MWA system of Covidien Emprint™ with Thermosphere™ Technology. The ablation antenna was placed inside the lesion under CT-guidance via 128-line multi-slice CT (Somatom Definition AS, Siemens) with the following settings: 5 mm fade-in, 30 mAs, 120 kV, 5 mm slice thickness, and activated real-time tube current modulation (CARE Dose 4D, Siemens). This targeted and careful approach could ensure a safe penetration and advancement of the antenna in the patient's body and reduced the risk of possible accidental injury to surrounding tissues. After a correct placement was confirmed, the ablation was initiated. During the procedure, repeated CT scans were acquired to monitor the procedure and to detect and react to any possible early complications. Adjustments to the antenna position could also be made to achieve the best possible therapeutic outcome. After a sufficient ablation time, the antenna is removed, and the entry channel is coagulated to reduce the risk of inadvertent distribution of tumor cells.

After the completion of the therapy, the patients were monitored for the following eight hours in case any complications occurred. If complications arose, such as a decrease in vigilance or a decline of vital functions, CT diagnostics were initiated to ensure that any possible complications of the ablation procedure such as bleeding could be detected and adequately treated. Complications were hereby differentiated into major and minor complications (Sacks et al. 2003).

### Transarterial chemoembolization

The patients included in this study received a cTACE in an outpatient setting.

After applying the local anesthetics, typically the femoral artery was punctured with a cannula via the Seldinger technique, in which initially a sheath is inserted into the vessel to create a sufficiently large access for the following catheters (Seldinger 2008), including a 5F Pig-Tail catheter (Boston Scientific) and a 5F Side-Winder catheter (Terumo, Tokyo, Japan). For this purpose, a contrast agent was injected after catheterization, to visualize and follow the further branching of the hepatic artery via the truncus celiacus to place the catheter directly into the tumor-supplying vessel. Several chemotherapeutic drugs were applied through the catheter, for instance, Mitomycin C Cisplatin as well as Irinotecan. The exact composition of the locally applied chemotherapeutics depended on the previously administered systemic chemotherapy regimen. After that, Lipiodol (Guerbet®) was administered into the vessel under fluoroscopic guidance. The embolization was concluded after a complete stasis of blood flow was achieved. After completion of the treatment, the catheters were removed and the incision point was covered with a compression bandage or a percutaneous closure device (Angio-Seal™, St. Jude Medical) as previously utilized (Gruber-Rouh et al. 2018). The post-ablation procedure did not differ from the MWA regimen, and the patients were discharged on the same day if no complications arose. In most cases, TACE was often repeated in several sessions. The response after TACE was assessed using the revised Response Evaluation Criteria in Solid Tumors (RECIST 1.1) (21).

### Imaging and follow-up

To evaluate the local tumor response and the course of the disease in the follow-up MR imaging, T1 sequences with and without contrast medium and T2 sequences were obtained, as well as diffusion-weighted sequences and In-phase and out-of-phase sequences. All examinations were performed on 1.5-T or 3-T MRI systems (Siemens).

### Tumor progression

Occurring tumor recurrences in the follow-up images were defined, as either LTP if the recurrence of tumor activity was located inside or adjacent to the ablation zone, if an initial complete ablation was achieved (Ahmed et al. 2014), or as a hDTP if the new hepatic lesion was not bordering the ablation zone.

### Statistical analysis

The OS was calculated from the date of the first MWA session until the last contact or death from any cause. The hPFS was calculated from the date of ablation until the first hepatic tumor progression or, if no progression occurred, to the date of the last follow-up or date of death. Both OS and hPFS were calculated using the Kaplan–Meier method and the log-rank test was used to compare survival between the groups. Continuous variables were compared using Mann–Whitney U test. Chi-square test was used to compare categorical variables.

A p-value of < 0.05 was defined as significant. All statistical calculations were made with the IBM® *SPSS*®-Software.

## Results

### Patients

The patients could be divided into two groups depending on the treatment protocol: Group A consisted of 184 patients [80 females (43.5%) and 104 males (56.5%); mean age: 64 ± 11.4 years], who underwent the combinatory treatment (TACE + MWA). Group B enrolled 67 patients [18 females (26.9%) and 49 males (73.1%); mean age: 63.2 ± 11.8 years], who were treated by MWA only and did not receive TACE.

A total of 442 metastases were treated in group A, with 67 patients having two (36.4%) and 68 patients (37%) having three or more treated metastases. Of these treated lesions 219 were below 2 cm in diameter (49.5%), 139 were two to three cm in diameter (31.5%) and the remaining 84 were 3 cm or larger in diameter (19%). The mean tumor diameter in group A was 2.3 ± 1.4 cm. The patients in group A received a total of 773 TACE sessions (mean 4.2 sessions/patient and mean 1.7 sessions/metastasis).

Group B on the other hand, had a total of 173 metastases treated, hereby 22 patients (32.8%) had two metastases treated and 25 three or more metastases treated (37.3%). Ninety-one (52.8%) of the lesions were below 2 cm in diameter, 59 (34.1%) between two and three centimeters and lastly 23 (13.1%) were equal to or above 3 cm in diameter. Continuing, for group B the average tumor diameter was 2.2 ± 1.3 cm. A summary of the patients’ and tumors’ characteristics can be seen in Table 1.

**Table 1:** Patients and tumor characteristics

	Group A	Group B	p-Value
Mean age	64 ± 11.4 years	63.2 ± 11.8 years	0.55
Sex			0.03
Female % (n)	43.5% (80)	26.9% (18)	
Male % (n)	56.5% (104)	73.1% (49)	
Number of metastases	442	173	0.16
One n (%)	26.6% (49)	29.9% (20)	0.98
Two n (%)	36.4% (67)	32.8% (22)	0.83
More than three n (%)	37.0% (68)	37.3% (25)	0.90
Diameter of metastases			0.62
< 2 cm n (%)	49.5% (219)	52.8% (91)	0.39
≥ 2-3 cm n (%)	31.5% (139)	34.1% (59)	0.85
≥ 3 cm n (%)	19.0% (84)	13.1% (23)	0.21
Mean tumor diameter	2.3 ± 1.4 cm	2.2 ± 1.3 cm	0.24

### Ablation

During all performed ablations 9 minor complications occurred, including local hemorrhage. Major complications did not occur in any of the patients.

**Fig. 1:**
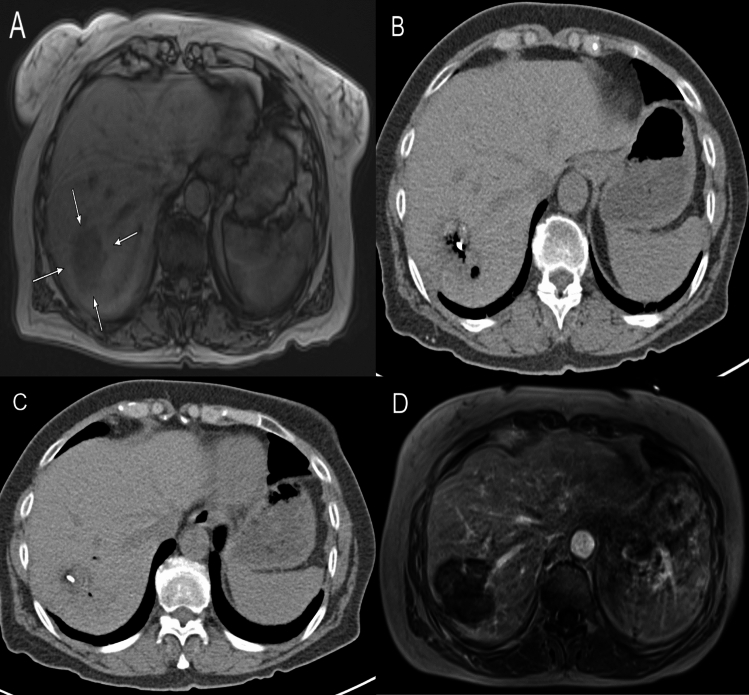
A patient with LMCRC treated by TACE followed by MWA. **A** MRI shows a LMCRC (white arrows) in the right liver lobe; **B,C** During MWA ablation, where some air bubbles can be seen; **D** MRI scan 24 h after MWA shows the ablation zone

In group A the mean diameter of the ablation zone 24 h post-ablation was 5.1 ± 1.4 cm. Herein, the average treatment duration was 9 ± 4.3 min and a mean energy of 46.2 ± 24.7 kJ was applied. As for the ablation parameters in group B, the mean ablation zone diameter was 4.9 ± 1.6 cm. The duration of ablations in group B were on average 9.3 ± 3.8 min. The average applied energy was 49.2 ± 24.1 kJ. An initial complete ablation could be achieved in 96.6% of cases (427/442) in group A, and in 96.5% of cases (167/173) in group B. A summary of the tumor and ablation parameters can be seen in Table 2. MWA procedure is shown in Fig. 1.

**Table 2:** Ablation parameters

	Group A	Group B	p-Value
Number of ablations	442	173	0.16
Mean diameter of ablation zone	5.1 ± 1.4 cm	4.9 ± 1.6 cm	0.039
Mean duration of MWA	9 ± 3.8 min	9.3 ± 3.8 min	0.61
Mean applied energy Incomplete ablations n (%) Minor complications n (%) Major complications n (%)	46.2 ± 24.7 kJ 15 (3.4) 5 (2.7) 0	49.2 ± 24.1 kJ 6 (3.5) 4 (6) 0	0.58 0.93 0.28 -

### Oncological outcome

All patients in group A achieved partial response or stable disease after TACE according to RECIST.

After MWA, the occurrence rate of LTP was 4.9% (9/184) in group A and 4.5% (3/67) in group B, (p-value: 0.062), and the rate of hDTP was 71.7% (132/184) in the combination therapy group and 83.6% (56/67) in the monotherapy group, (p-value: 0.81).

### Survival analysis

The statistical analysis found a median OS of 30 months (95% CI 26–34) for group A and 26 months (95% CI 18–34) for group B. However, this difference was not significant (p-value: 0.67). The one-, two-, three- and four-year OS rates were 84.2%, 61.1%, 40.8%, and 31.3% for group A, and 82.3%, 53.2% 34.6%, and 28.2% for group B, respectively. Hereby, the mean time to hPFS was 13.8 months (95% CI 10.9–16.8) for group A and 8.1 months (95% CI 6.1–10.1) for group B. With a p-value of 0.03 for the hPFS time, a significant difference could be found between both groups. The 6-, 12- and 24-month hPFS OS rates were 43.6%, 28.6%, and 15.6%, for group A, and 38.6%, 14.7%, and 5.5% for group B, respectively.

**Fig. 2:**
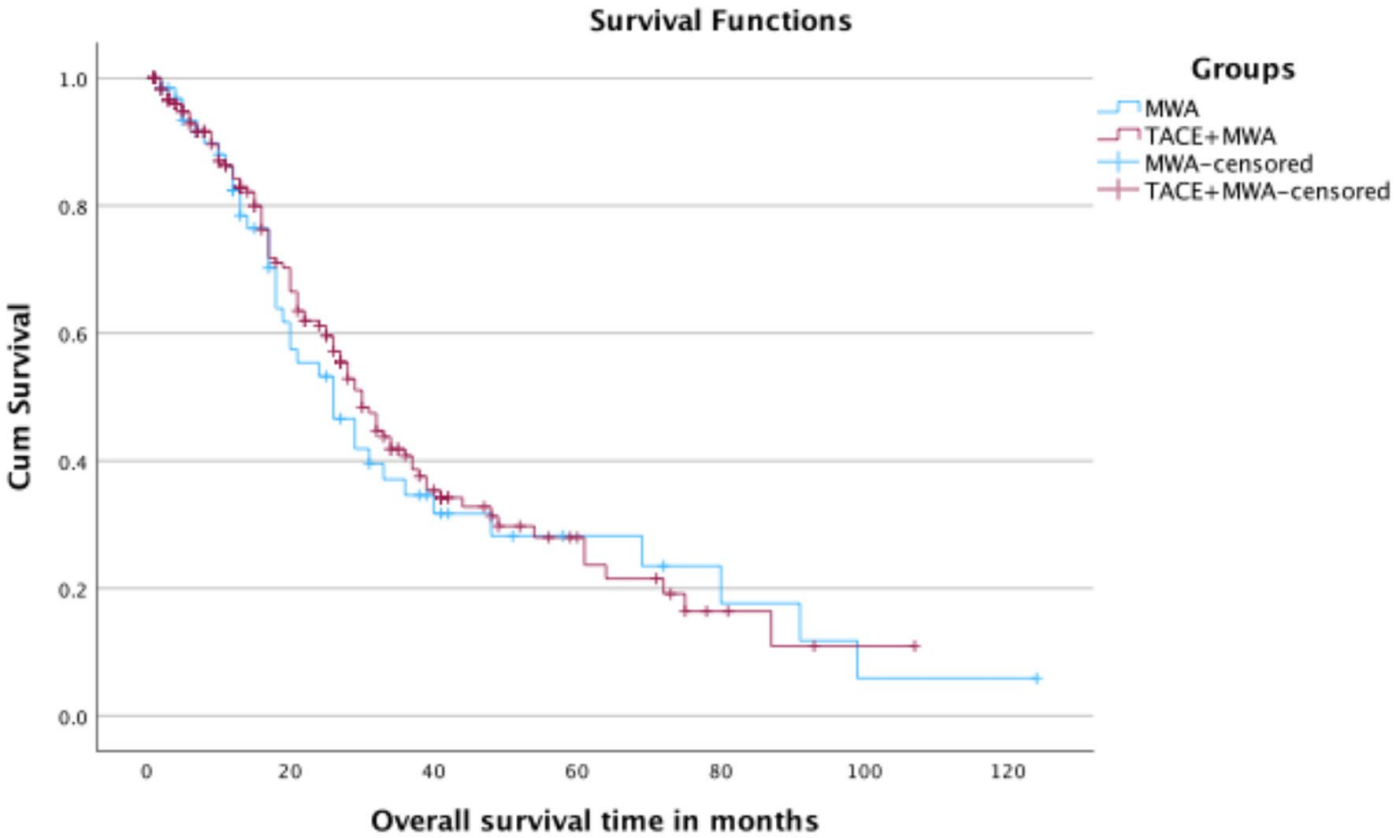
Kaplan–Meier curves of OS for both groups

**Fig. 3:**
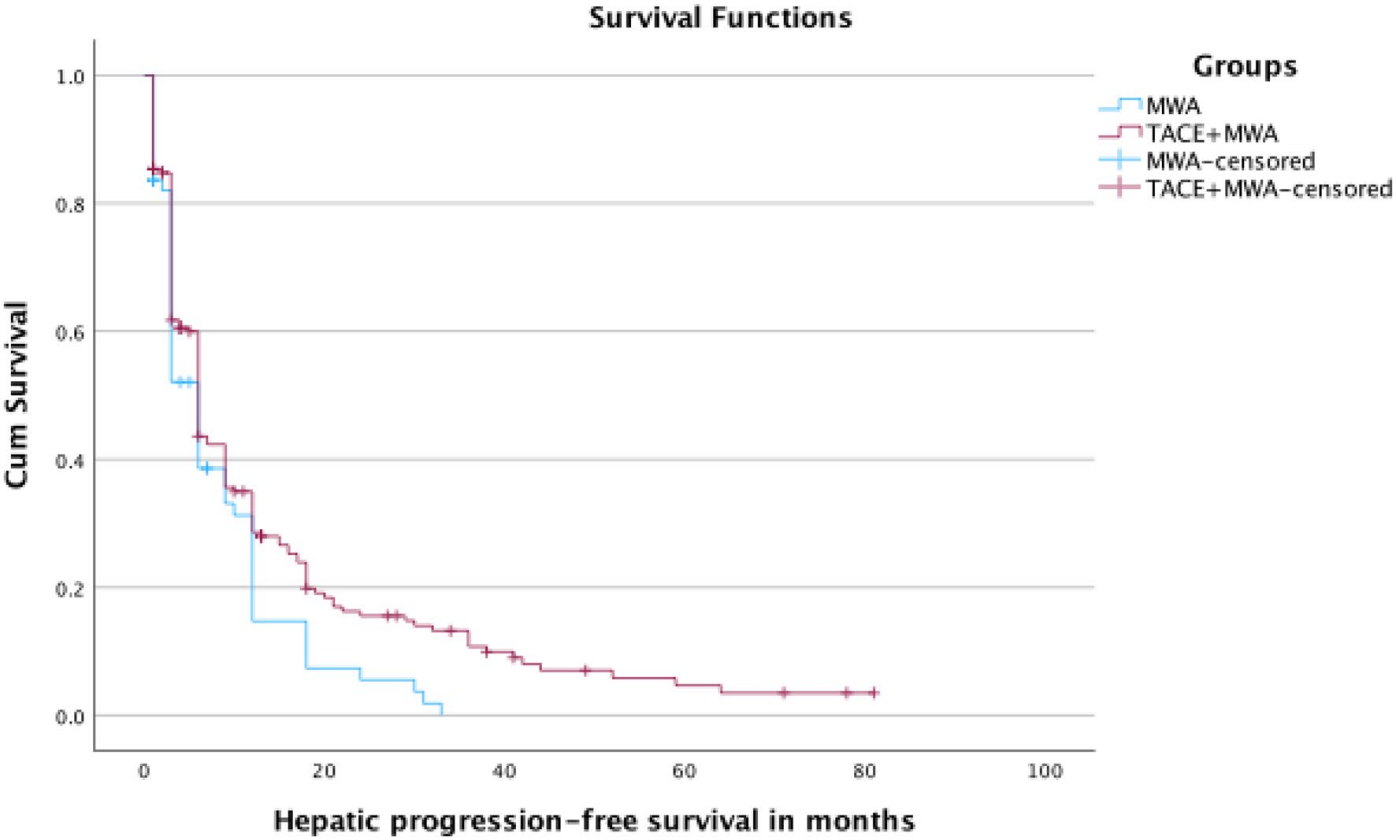
Kaplan–Meier curves of hPFS for both groups

Survival analysis is summarized in Table 3. Kaplan–Meier curves of OS and hPFS are shown in Figs. 2 and 3, respectively.

**Table 3:** Survival

	TACE + MWA	MWA	p-Value
Median OS (months)	30 (95% CI: 26–34)	26 (95% CI: 18–34)	0.67
1-year OS rate	84.2%	82.3%	
2-year OS rate	61.1%	53.2%	
3-year OS rate	40.8%	34.6%	
4-year OS rate	31.3%	28.2%	
Mean hPFS (months) 6-month hPFS rate 12-month hPFS rate 24-month hPFS rate	13.8 (95% CI: 10.9–16.8) 43.6% 28.6% 15.6%	8.1 (95% CI: 6.1–10.1) 38.6% 14.7% 5.5%	0.03

## Discussion

This retrospective study aimed to analyze whether a combination of TACE and MWA may provide a beneficial impact on the outcome of patients with LMCRC, in comparison to a treatment by MWA alone. The patients were divided into two groups depending on which therapy they received. Both groups were similar in age, number, and size of treated metastases, ablation zone size, duration of MWA, and applied energy, therefore a valid comparison can be made. However, the two groups varied in the total number of included patients, with group A having more than double the number of patients than group B.

It was shown that the combination of both therapies provides a non-significant better OS and significantly longer hPFS, in comparison to monotherapy. The lower rate of hDTP in the combination group may be the reason for the longer OS, despite the non-significant differences between both groups.

The theory was that a preceding TACE therapy would decrease the vitality of the metastases due to damaged vascularisation and local application of chemotherapeutics, which would then lead to shrinkage in size and facilitate the following MWA (Liu et al. 2020; Vogl et al. 2007). We could show that the MWA after TACE required less energy application as well as less ablation time, but provided significantly larger ablations zones compared to MWA as monotherapy.

TACE was introduced in the 1970s by Yamada et al. and was mainly thought of as a second-line treatment for unresectable tumors, after failure of systemic chemotherapy (Gruber-Rouh et al. 2016; Yamada et al. 1983). However, in combination with other locoregional therapies, such as MWA, TACE could be discussed as a first-line treatment for hepatic lesions, given the fact that TACE can provide a sufficient downsizing of these lesions of up to -35% in volume, ensuring a small enough tumor for complete ablation by MWA (Vogl et al. 2003). Therefore, it may be possible to treat lesions larger than 3 cm, given the fact that the efficacy of ablative therapies alone significantly decreases in tumors larger than 3 cm (Seager et al. 2021; Acciuffi et al. 2022). For instance, a study by Kobe et al. has shown that a combination of TACE and ablative therapies in liver metastases larger than 3 cm in diameter is a safe technique with good local tumor control (Kobe et al. 2022).

On the other hand, our study could not show a significant advantage for the combination therapy regarding the hepatic tumor response. In our study, group A had a slightly higher LTP rate of 4.9% than the monotherapy group B with 4.5%. However, the LTP rate achieved in this study is similar to those of other studies (Groeschl et al. 2014; Stättner et al. 2013). The hDTP rates however differed between the two groups in our study, with a hDTP rate of 83.6% in group B and a lower rate of 71.7% in group A, this may be because of the reduced vascularization and vitality of the tumor after TACE. Therefore, the ability of the tumor to spread and generate new hepatic tumor loci may be inhibited.

This study was able to show that the combination of both therapies leads to a significantly better hPFS and can be interpreted as a success in proving that devascularization of the tumor before ablative treatment can produce better local tumor control (Li and Ni 2019).

In comparison, a study by Fong et al. was able to show that a significant impact on OS can be achieved. The patients had a 1-year OS of 93.8% and a 3-year OS of 50% with a combination therapy consisting of TACE and different ablative therapies, such as MWA, RFA, and cryoablation (Ven Fong et al. 2012). The study included fewer patients than ours, but 47% of patients had only one hepatic lesion, in comparison to 26.6% in our study (Ven Fong et al. 2012). Therefore, it may be possible that this led to a better OS because the number of hepatic lesions is a prognostic factor for hepatic recurrence and liver function (Liang et al. 2003).

Regarding intraprocedural safety, there were no major complications reported. However, the rate of minor complications was higher in the monotherapy group compared to the combination group without a significant difference. Post-ablation complications were mainly local hemorrhages, without any severe following consequences. The devascularization caused by TACE may be the reason for the lower rate of hemorrhages in the combination group. Though MWA per se is an intervention with few complications as proven before (Izzo et al. 2019; Lahat et al. 2014). Other adverse side effects connected to MWA or TACE did not occur. Given the fact that TACE and MWA are minimally invasive procedures with few complications, it can be ideally applied in patients with inoperable metastases, or patients unable to undergo general anesthesia and surgery.

Lastly, this study has several limitations. For one, the retrospective character of this study. The data was collected after the completion of the therapies and subsequently analyzed and evaluated. Moreover, the patients’ cohorts show a comparatively high quantity but remains inhomogeneous compared to other studies with more similar study populations. Patients in curative and palliative therapy management were not differentiated in this study. Moreover, no data were available on CRC mutations such as RAS or BRAF, therefore the influence of these on the subsequent prognosis remains unclear. Finally, prospective randomized studies are required for a better comparison of both treatments protocols.

## Conclusion

In summary, this study has proven that MWA is a safe and effective minimally invasive therapy for LMCRC, and TACE as a preliminary therapy can enable better local tumor control and significantly prolong the hPFS. However, more extensive research is necessary to also prove a beneficial impact on OS, as this could not be significantly increased in this study. Nevertheless, a combination of TACE and MWA should be discussed in patients, who are not responding to systemic chemotherapy and unable to undergo surgery for LMCRC.

## Data Availability

The datasets generated during and/or analysed during the current study are available from the corresponding author on reasonable request.
